# Phenotype and genotype analysis for *Helicobacter pylori* antibiotic resistance in outpatients: a retrospective study

**DOI:** 10.1128/spectrum.00550-23

**Published:** 2023-09-21

**Authors:** Mengqiu Xiong, Hend Sadeq Mohammed Aljaberi, Nida Khalid Ansari, Yalan Sun, Sijie Yin, Lubanga Nasifu, Huiling Sun, Tao Xu, Yuqin Pan, Zhenlin Nie, Caidong Liu, Zhenyu Zhang, Zongdan Jiang, Shukui Wang, Bangshun He

**Affiliations:** 1 Department of Laboratory Medicine, Nanjing First Hospital, Nanjing Medical University, Nanjing, China; 2 School of Basic Medicine and Clinical Pharmacy, China Pharmaceutical University, Nanjing, China; 3 Department of Laboratory Medicine, Yangzhou Hongquan Hospital, Yangzhou, China; 4 General Clinical Research Center, Nanjing First Hospital, Nanjing Medical University, Nanjing, China; 5 Department of Gastroenterology, Nanjing First Hospital, Nanjing Medical University, Nanjing, China; 6 H. pylori Research Key Laboratory, Nanjing Medical University, Nanjing, China; Tainan Hospital, Ministry of Health and Welfare, Tainan, Taiwan

**Keywords:** antibiotic resistance, genotype, *Helicobacter pylori*, outpatients, phenotype

## Abstract

To investigate the antibiotic resistance of *Helicobacter pylori* (*H. pylori*) in outpatients and to explore the consistency between genotype and phenotype of *H. pylori* antibiotic resistance. A retrospective study on outpatients screened with urea breath test for *H. pylori* infection in Nanjing First Hospital from April 2018 to January 2022. Patients who tested positive underwent a consented upper endoscopy, and the *H. pylori* infection was confirmed by rapid urease test (RUT) and *H. pylori* culture. For antibiotic resistance phenotype analysis, the *H. pylori* strains isolated from gastric biopsy were tested for antibiotic resistance phenotype by the Kirby-Bauer disk diffusion test. In addition, the antibiotic resistance genotype of isolated *H. pylori* was tested with a real-time polymerase chain reaction. A total of 4,399 patients underwent *H. pylori* infection screening, and 3,306 *H. pylori* strains were isolated. The antibiotic resistance phenotype test revealed that the resistance rates of metronidazole (MTZ), clarithromycin (CLR), levofloxacin (LEV), amoxicillin (AMX), furazolidone (FR), and tetracycline (TE) were 74.58%, 48.61%, 34.83%, 0.76%, 0.27%, and 0.09%, respectively. Additionally, the antibiotic resistance genotype test revealed that rdxA gene mutation A610G (92.96%), A91G (92.95%), C92A (93.00%), and G392A (95.07%) were predominant in *H. pylori* with MTZ resistance; 23S rRNA gene mutation A2143G (86.47%) occurred in most *H. pylori* with CLR resistance; and gyrA gene mutation 87Ile/Lys/Tyr/Arg (97.32%) and 91Asn/Gly/Tyr (90.61%) were the most popular mutations in strains with LEV resistance. The phenotypic resistance and genotypic resistance to CLR (kappa value = 0.824) and LEV (kappa value = 0.895) were in good agreement. The history of eradication with MTZ, CLR, LEV, and AMX was correlated with *H. pylori* resistance. In short, this study demonstrated that drug resistance of *H. pylori* was mainly to MTZ, CLR, and LEV in local outpatients. Three drugs can be selected for increased MICs (Minimum Inhibitory Concentration) via single chromosomal mutations. In addition, the genotype could be used to predict the phenotypic *H. pylori* resistance to CLR and LEV.

**IMPORTANCE:**

*Helicobacter pylori* is a key bacterium that causes stomach diseases. There was a high prevalence of *H. pylori* in the Chinese population. We analyzed the resistance phenotype and genotype characteristics of *H. pylori* in 4,399 outpatients at the First Hospital of Nanjing, China. We found a higher resistance rate to metronidazole (MTZ) , clarithromycin (CLR), and levofloxacin (LEV), and the genotype could be used to predict the phenotypic *H. pylori* resistance to CLR and LEV. This study provides information on *H. pylori* infection and also provides guidance for clinical doctors' drug treatment.

## INTRODUCTION

*Helicobacter pylori* (*H. pylori*) is the main pathogenic factor of various gastric diseases, including gastric cancer. Epidemiological studies have shown that almost half of the individuals worldwide have *H. pylori* infection, especially in developing countries, which seriously threatens human health (1). According to published data, the average *H. pylori* infection rate in China is 58.07% (2). The positive ratio of *H. pylori* infection varies with region and age (3). Most patients were primarily infected with *H. pylori* as children, with long-term concealment eventually leading to various disorders (4).

Presently, *H. pylori* infection detection methods are classified as invasive and non-invasive. The invasive detection methods include endoscopy imaging, rapid urease test (RUT), histology, culture, and molecular methods of endoscopic biopsy specimens. The non-invasive detection methods include the urea breath test (UBT), stool antigen test, serological, and molecular examinations. Each method for diagnosing *H. pylori* has both advantages and disadvantages (5).

*H. pylori* infection can lead to the occurrence of many diseases. The disease itself cannot be resistant to antibiotics, and the human body itself cannot produce drug resistance. However, different strains of bacteria change the genetic material through different methods and make themselves resistant to antibiotics; so that the bacteria cannot be degraded by the antibiotics, thereby losing the bactericidal effect. This makes it impossible to eradicate the disease. *H. pylori* eradication was suggested by many countries, such as the Chinese consensus currently recommends a quadruple combination of bismuth [proton pump inhibitor (PPI) + bismuth + two antibiotics] as the primary empirical eradication regimen (6). There were several recommended consensuses as follows: (i) the Maastricht V/Florence consensus that pointed out that the resistance rate of *H. pylori* to antibiotics is increasing in most parts of the world (7); (ii) the Houston consensus that recommends treatment for patients with active *H. pylori* infection and suggests individuals of Latino and African-American ethnicity should consider *H. pylori* testing (8); and (iii) the 2015 Kyoto consensus that initiated the march to eliminate *H. pylori* for the first time: all *H. pylori* positives should be eradicated (9). The remedy for *H. pylori* eradication in the clinical setting was constantly modified according to clinical outcomes, such as first-line triple therapy. However, they are ineffective in more than 20% of patients, mainly due to the increasing resistant strains of one or more antibiotics used in these therapies (10). According to Maastricht V consensus suggestion, bismuth-containing quadruple therapy was recommended as the first-line treatment in regions where the clarithromycin (CLR) resistance rate exceeded 15%–20% (11). However, the decline in the eradication rate of *H. pylori* was due to multiple factors, including increased antibiotic resistance rate, low patient compliance, high bacterial load (5), and genotype of cytochrome P450 proteins 2C19 (CYP2C19) gene (7). The increased antibiotic resistance due to *H. pylori* gene mutation has been a broad concern among them. Herein, the detection of antibiotic resistance related to *H. pylori* gene mutation was widely applied for *H. pylori* precise eradication. CLR resistance is related to a single-nucleotide substitution on the 23S rRNA gene of *H. pylori*, herein, which the detection of this nucleotide substitution can predict. Also, the clarithromycin resistance of *H. pylori* could be determined by using cultured *H. pylori*, the cleaved amplification polymorphism sequence-tagged sites (PCR-Restriction Fragment Length Polymorphism) method, SELEX Affinity Landscape Mapping PCR (SELMAP-PCR) method, the direct sequencing method, or the single-nucleotide primer extension method (12). In addition, various molecular methods, including PCR, have been used to detect the resistance of *H. pylori* to CLR (13).

However, the correlation between genotypic and phenotypic *H. pylori* antibiotic resistance strains in China is not yet fully elucidated. Therefore, we try to verify their consistency guide eradication therapy further. This article aims to investigate the antibiotic resistance of *H. pylori* in Nanjing, Jiangsu, China, explore the consistency of antibiotic-resistance genotypes and phenotypes, predict *H. pylori* antibiotic resistance by antibiotic resistance genotype detection, and improve eradication ratio for *H. pylori* infected patients.

## MATERIALS AND METHODS

### Patients and samples

Outpatients who visited Nanjing First Hospital, Nanjing Medical University for their gastrointestinal symptoms from April 2018 to January 2022 were included in the study for their positive results of the ^13^C-UBT. The clinical records were retrieved by screening case records. Written informed consent was obtained from all of the participants.

### Culture of *H. pylori* and antibiotic resistance phenotype testing

During routine gastroscopy, a clinician uses sterile biopsy forceps to remove gastric mucosal tissue from the greater or lesser curvature 2–3 cm anterior to the pylorus, and then the *H. pylori* strain is inoculated into a Colombian blood plate and cultured in an incubator containing 5% O_2_, 10% CO_2_, and a temperature of 37°C for 3–5 days. Finally, the *H. pylori* strains were confirmed by Gram staining and RUT.

The Kirby-Bauer disk diffusion test (K-B test) was used to determine the antibiotic resistance phenotype. Specifically, *H. pylori* strains were cultured in M-H agar plate medium (Guangdong Huankai Microbial Sci. & Tech. Co., Ltd) containing 10% sheep blood or Columbia blood plate medium (Kemajia Microbe Technology Co., Ltd). After the plate was dried, sterilized tweezers were used to affix the antibiotic-sensitive paper (OXOID/Thermo) to the plate (three per plate). The resistance criteria for the zone of inhibition were defined as CLR ≤ 13 mm, tetracycline (TE) ≤14 mm, levofloxacin (LEV) < 13 mm, furazolidone (FR) ≤ 14 mm, metronidazole (MTZ) < 16 mm, and amoxicillin (AMX) < 14 mm. According to the updated European Committee on Antibiotic Susceptibility Testing (EUCAST) recommendations, MIC values of 0.5 and 8 mg/L are the cut-offs above which *H. pylori* is deemed resistant to CLR and MTZ, respectively (14). In this experiment, we determine whether it is resistant by the size of the inhibition circle formed by the drug content of the drug-sensitive paper sheet.

### Antibiotic resistance genotype testing

*H. pylori* DNA was extracted from the gastric mucosal specimen using the DNA extraction kit (HiPure Blood & Tissue DNA Mini Kit Universal column, Magen Biotech, Guangzhou, China). To identify resistance via conventional PCR-based analysis, the DNA region, including the mutations (23S rRNA, gyrA, PBP1A, porD, oorD, 16S rRNA, and rdxA), was amplified by PCR. Then the PCR products were sequenced using Sanger sequencing, as previously described (15).

### Statistical analysis

SPSS 22.0 software was used for data analysis. The normally distributed data are expressed as *x* ± *s*; independent samples *t*-test is used for comparison between groups; kappa consistency test is used to analyze the consistency of antibiotic resistance genotypes and phenotypes; antibiotic resistance rate was compared with χ test; and *P* < 0.05 was considered statistically significant.

## RESULTS

### Basic characteristics of enrolled outpatients and *H. pylori* screening results

**Fig 1:**
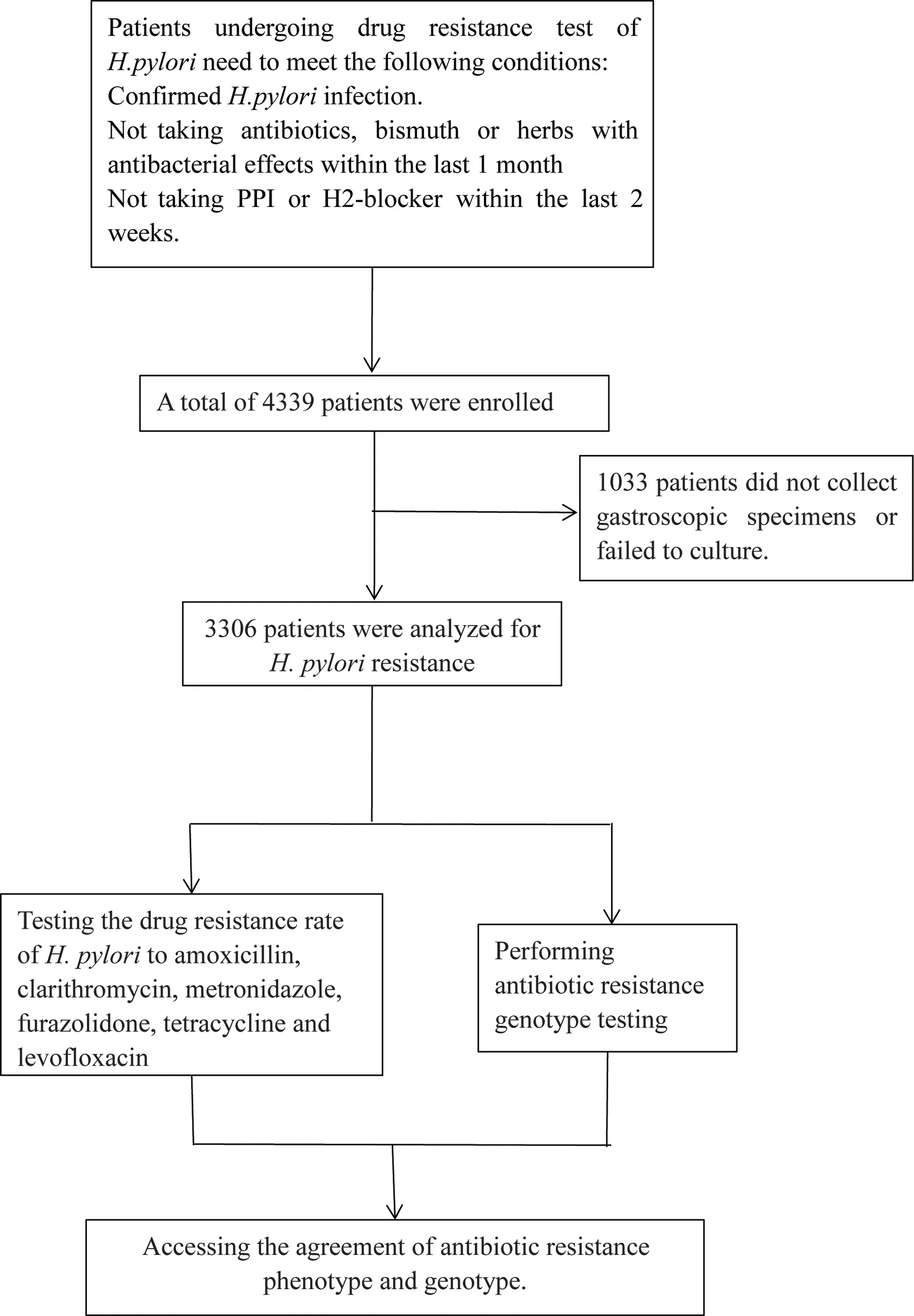
Flowchart depicting the study design.

A total of 4,399 outpatients with positive *H*. *pylori* infection by ^13^C-UBT or RUT were enrolled in this study, and 4,255 *H*. *pylori* strains were isolated from patients. Out of them, a total of 3,306 strains of *H. pylori* from patients were analyzed for antibiotic resistance phenotypes, and 949 patients who failed to yield culture were eventually excluded from the phenotype analysis (Fig. 1). Clinical information for a total of 4,327 patients was retrieved from all enrolled patients, consisting of 2,206 males and 2,121 females, whose average ages were 47.56 ± 13.59 and 47.70 ± 13.02 years old, respectively. Most patients were diagnosed to have gastritis (92.43%), duodenal ulcer (11.43%), esophagitis (6.62%), and gastric ulcer (4.57%) (Table 1).

**TABLE 1:** Baseline characteristics of study cohort

Characteristics	Overall (*n* = 4,327)
Gender	
Male	2,206
Female	2,121
Age, mean (SD)	
Male	47.56 ± 13.59
Female	47.70 ± 13.02
Gastroscopy results	
Gastritis	4,066 (92.43%)
Gastric ulcer	201 (4.57%)
Duodenal ulcer	503 (11.43%)
Esophagitis	291 (6.62%)

Among the successfully isolated 3,306 *H*. *pylori* strains, we combined the result of *H. pylori* culture, ^13^C-UBT, and RUT. The results showed that the positive rate of ^13^C-UBT and RUT was 99.66% and 91.65%, respectively (Table 2).

**TABLE 2:** Analysis of *H. pylori* diagnostic results of breath test and rapid urease test

Method	*H. pylori* culture		
		Positive, ***n*** (%)	Negative, ***n*** (%)
Urea breath test	Positive	1,172 (99.66）	371 (96.87）
	Negative	4 (0.34）	12 (3.13）
	Overall	1,176	383
Rapid urease test	Positive	3,009 (91.65）	721 (77.19）
	Negative	274 (8.35）	213 (22.81）
	Overall	3,283	934

### Antibiotic resistance phenotype

The antibiotic resistance rate of *H. pylori* to antibiotics was detected by the K-B test for those *H. pylori* strains successfully isolated patients. The results showed that MTZ, CLR, LEV, AMX, FR, and TE resistance rates were 74.58%, 48.61%, 34.83%, 0.76%, 0.27%, and 0.09%, respectively (Table 3).

**TABLE 3:** Results of antibiotic resistance phenotype

Antibiotics	*n*	Sensitive, *n* (%)	Resistant, *n* (%)
MTZ	3,301	839 (25.42)	2,462 (74.58)
CLR	3,304	1,698 (51.39)	1,606 (48.61)
LEV	3,299	2,150 (65.17)	1,149 (34.83)
AMX	3,306	3,281 (99.24)	25 (0.76)
FR	3,305	3,296 (99.73)	9 (0.27)
TE	3,306	3,303 (99.91)	3 (0.09)

### Antibiotic resistance genotype

For all tested antibiotics, the genotypes correlated with antibiotic resistance were also analyzed by using PCR in biopsy specimens (Table 4), and the results showed that the rates of antibiotic resistance genotypes of CLR, MTZ, and LEV were relatively high. Among the genotypes, the predominated mutation-related CLR was A2143G (86.47%), MTZ was A610G (92.96%), A91G (92.95%), C92A (93.00%), G392A (95.07%), and LEV was 87Ile/Lys/Tyr/Arg (97.32%) and 91Asn/Gly/Tyr (90.61%). The relationship between the multi-antibiotic resistance gene mutations and antibiotic resistance outcomes was investigated to examine the alteration of the resistance phenotype by multiple mutations. Among high resistance rate antibiotics, MTZ and LEV were the two antibiotics giving rise to more complicated mutation sites, indicating that the proportion of drug resistance caused by multi-site mutation was significantly higher than that caused by single drug resistance gene mutation (Table S1). To investigate the consistency of the genotype and phenotype of antibiotic resistance of *H. pylori*, a kappa consistency test was carried out for all tested six antibiotics, and the result revealed that phenotypic and genotypic resistance to CLR (kappa value = 0.824) and LEV (kappa value = 0.895) were in good agreement (Table S2).

**TABLE 4:** *H. pylori* antibiotic resistance genotypes

Antibiotics	Gene	Mutation	*n*	Sensitive (%)	Resistant (%)
CLR					
1	23S rRNA	A2143G	1,774	240 (13.53）	1,534 (86.47）
2		A1141G	6	0 (0）	6 (100）
MTZ					
1	rdxA	A610G	611	43 (7.04）	568 (92.96）
2		A61G	99	18 (18.18）	81 (81.82）
3		T62C	113	20 (17.70）	93 (82.30）
4		A91G	611	43 (7.05）	568 (92.95）
5		C92A	614	43 (7.00）	571 (93.00）
6		G392A	548	27 (4.93）	521 (95.07）
7		A614C	3	0 (0）	3 (100）
LEV					
1	gyrA	87Ile	34	1 (2.94）	33 (97.06）
2		91Asn	75	12 (16.00）	63 (84.00）
3		87Lys	254	7 (2.76）	247 (97.24）
4		91Gly	69	4 (5.80）	65 (94.20）
5		91Tyr	37	1 (2.70）	36 (97.30）
6		87Tyr	8	0 (0）	8 (100）
7		87Arg	2	0 (0）	2 (100）
8		87Ile/Lys/Tyr/Arg	298	8 (2.68）	290 (97.32）
9		91Asn/Gly/Tyr	181	17 (9.39）	164 (90.61）

### History of *H. pylori* eradication and antibiotic resistance phenotypes

**Fig 2:**
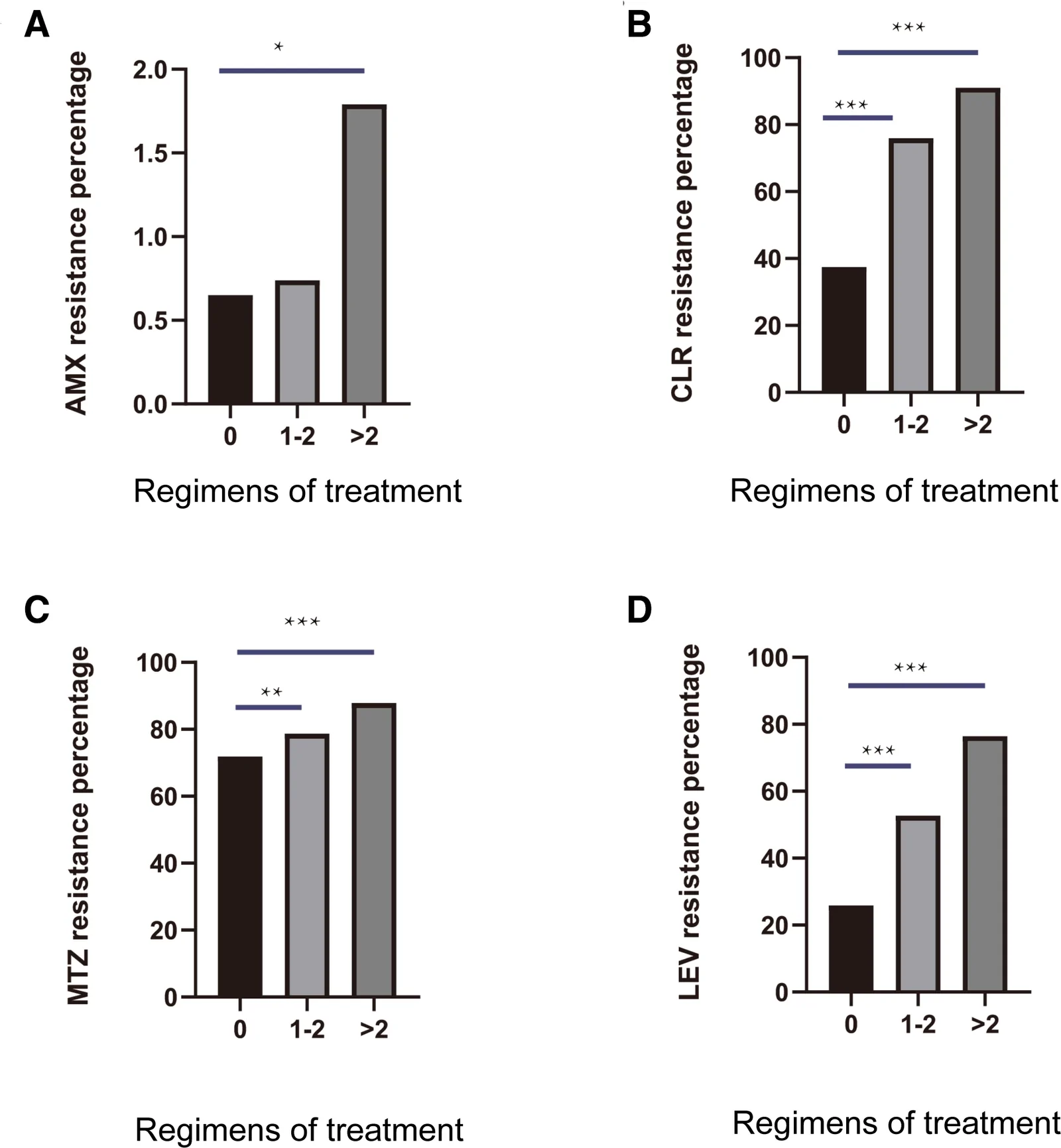
The association of *H. pylori* antibiotic resistance and the times of treatments. *P < 0.05, **P < 0.01, and ***P < 0.001.

Outpatients in the analysis included 2,461 untreated cases, 541 had already received treatment once or twice, and 280 had received treatment in multiple regimens. In order to understand the effect of the history of *H. pylori* eradication on the antibiotic resistance phenotype, we grouped the antibiotic-resistant patients according to the regimens of treatment (Fig. 2). In the gene mutation subgroup of MTZ, CLR, LEV, and AMX, the mutation rate significantly increased in treated patients compared with untreated patients.

## DISCUSSION

This retrospective study included 4,399 outpatients to investigate the phenotypes and genotypes of *H. pylori* antibiotic resistance and their consistency. Our results revealed that the antibiotic resistance rate of MTZ (74.58%), CLR (48.61%), and LEV (34.83%) was relatively high, that the antibiotic resistance and genotype of CLR and LEV were highly consistent, and that eradication times of *H. pylori* were significantly correlated with the rate of antibiotic resistance.

In this study, ^13^C-UBT and RUT were initially used to screen and confirm for *H. pylori* infection, respectively. We observed that the positive rate of ^13^C-UBT was higher than that of RUT, indicating ^13^C-UBT is a valuable method for *H. pylori* infection screening. In contrast, RUT is not an excellent method for *H. pylori* infection diagnosis, consistenting with previously reported results (16), which was attributed to that RUT is based on the urease reaction of gastroscopic specimens, a lower sensitive method for *H. pylori* detection, and that it takes minutes to hours to provide results, which could be affected by the sampling of gastroscopic specimens and a certain kind of commercial RUT kit used (17). Currently, the detection of *H. pylori* infection mainly includes serum antibody detection, breath test, gastroscopy to mucosal RUT, and monoclonal stool antigen test. However, these laboratory diagnostic methods have different shortcomings. Existing studies have shown that stool-based *H. pylori*-PCR has good diagnostic performance, but the positive rate is often affected by the amplification of the target gene (18). Some studies have shown that slot PCR has high sensitivity and is considered the gold standard for the diagnosis of *H. pylori*.

The antibiotic resistance of *H. pylori* in the local area shows that the antibiotic resistance rate of MTZ, CLR, and LEV is relatively high, which was consistent with previously reported results that, in China, the antibiotic resistance rate was 77%, 37%, and 33% for MTZ, CLR, and LEV, respectively (19). In view of this situation, treatment regimens with MTZ should be avoided, and the choice of CLR and LEV used to treat patients should be adjusted accordingly. The precise eradication of *H. pylori* should be carried out based on the fact that, in China, standard triple therapy containing PPI, CLR, and AMX (or MTZ) is often used as the first-line therapy for eradication.

Since the discovery of drug resistance genes, many genes for *H. pylori* have been studied. In 1996, Versalovic et al. detected a 23S RNA gene point mutation responsible for CLR resistance in *H. pylori* strains in domain V (20). Today, these mutations are known as A2143G and A2142G. In our study, A2143G is the major mutant genotype of CLR. The key mechanism leading to *H. pylori* MTZ resistance is a null mutation in the chromosomal rdxA gene encoding oxygen-insensitive NADPH (nicotinamide adenine dinucleotide phosphate) nitroreductase (21). Miyachi found that the mechanism of LEV resistance is associated with mutations in the gyrA gene in the quinolone resistance determinant region (22). In this study, we also found a strong link between these drug-resistant gene mutations and phenotypes. There are currently many studies that sequence the complete genome of pathogens and offer more complete information, which explains the poor concordance between phenotypic and genotypic data. Therefore, there are still limitations in this paper, and it is hoped that latest techniques can be applied to explore the relationship between genotype and phenotype in future studies.

The failure of *H. pylori* eradication therapy could be attributed to extensive antibiotic resistance, changes in the virulence of *H. pylori* strains, CYP2C19 gene polymorphism, and poor patient compliance. Antibiotic resistance is the primary factor in the decline of the eradication rate (23). For the precise eradication of *H. pylori*, the antibiotic sensitivity assessment for *H. pylori* is critical for designing and optimizing the most effective therapy (24). Herein, the analysis for antibiotic resistance of phenotype, which was based on isolated *H. pylori* culture, and the analysis for antibiotic resistance of genotype, which was based on isolated *H. pylori* antibiotic-associated mutation detection, were two optimal ways for eradication therapy design (16). In this study, we assessed the consistence of phenotype and genotype of six antibiotic resistence to *H. pylori*. Our result revealed that the genotype was consistent with the phenotype for antibiotics of CLR and LEV, indicating that the genotype of *H. pylori* antibiotic resistance analysis could be used for eradication therapy design.

In this retrospective study, we found that the times *H. pylori* eradication was correlated with antibiotic resistance phenotypes, suggesting that *H. pylori* eradication led to the occurrence of antibiotic resistance or the failure of *H. pylori* eradication was attributed to the presence of antibiotic resistance. Herein, the susceptibility guide treatment effectively achieves high efficacy with limited side effects and avoids unnecessary antibiotic use (25). Therefore, the genotype or phenotype detection for *H. pylori* antibiotic resistance is a promising method for the precise eradication of *H. pylori*.

There are still some shortcomings in this article. First, the purpose of this study was to investigate the consistency of the genotype and phenotype of *H. pylori* antibiotic resistance based on the obtained genotype information irrespective of the sub-strains. Admittedly, the mixed infection has a potential effect on the consistency of the genotype and phenotype, which was a limitation of this study. With in-depth research on *Helicobacter pylori*, in this study, we selected several previously identified mutational sites related to drug resistance. Admittedly, due to the limitation of the method applied for genotype analysis, we could not explore all potential mutational sites, which could have led to the reduced concordance of the genotype and phenotype.

In short, this study demonstrated that MTZ, CLR, and LEV were the main resistant antibiotics in local outpatients. The genotype could be used to predict the antibiotic resistance of CLR and LEV.

## SUPPLEMENTAL MATERIAL

Supplemental tables spectrum.00550-23-s0001.docx Table S1 and Table S2.

ASM does not own the copyrights to Supplemental Material that may be linked to, or accessed through, an article. The authors have granted ASM a non-exclusive, world-wide license to publish the Supplemental Material files. Please contact the corresponding author directly for reuse.
