# Supplementary material for: Phenotype and genotype analysis for Helicobacter pylori antibiotic resistance in outpatients: a retrospective study
Source: Microbiol Spectr. 2023 Sep 21;11(5):e00550-23. doi: 10.1128/spectrum.00550-23 (PMC10580949; doi:10.1128/spectrum.00550-23)
Supplement: Supplemental tables — Table S1 and Table S2. [file spectrum.00550-23-s0001.docx]

**Supplementary Table 1: Genotypes of *H.pylori* multiple drug resistance**

| Antibiotics | Number of mutations | Multiple gene mutations* | n | % |
| --- | --- | --- | --- | --- |
| MTZ | Two | 4；5 | 3 | 100 |
|  |  | 1；6 | 9 | 100 |
|  | Three | 2；3；6 | 1 | 100 |
|  |  | 1；4；5 | 40 | 90.91 |
|  |  | 4；5；6 | 13 | 92.86 |
|  |  | 1；4；6 | 1 | 100 |
|  |  | 1；5；6 | 2 | 100 |
|  | Four | 1；4；5；6 | 412 | 95.81 |
|  |  | 1；2；3；5 | 1 | 100 |
|  |  | 1；3；4；5 | 1 | 100 |
|  | Five | 1；2；3；4；5 | 14 | 53.85 |
|  |  | 2；3；4；5；6 | 2 | 100 |
|  |  | 1；2；3；5；6 | 1 | 100 |
|  |  | 1；2；4；5；6 | 6 | 100 |
|  |  | 1；3；4；5；6 | 14 | 93.33 |
|  |  | 1；4；5；6；7 | 3 | 100 |
|  | Six | 1；2；3；4；5；6 | 59 | 89.39 |
| LEV | Two | 1；2 | 1 | 100 |
|  |  | 1；5 | 2 | 100 |
|  |  | 2；3 | 4 | 100 |
|  |  | 2；4 | 4 | 57.14 |
|  |  | 2；5 | 2 | 100 |
|  |  | 1；3 | 3 | 100 |
|  |  | 3；4 | 12 | 100 |
|  |  | 3；5 | 4 | 100 |
|  |  | 4；5 | 4 | 100 |
|  |  | 6；2 | 1 | 100 |
|  |  | 6；3 | 1 | 100 |
|  | Three | 2；4；5 | 1 | 100 |

* Number were adopted from Table 4

**Supplementary Table 2. Agreement between phenotypic and genotypic resistance**

| Antibiotics | Genotype | Phenotype | | Accordance rate | Kappa Value | P value |
| --- | --- | --- | --- | --- | --- | --- |
|  |  | Resistant | Sensitive |  |  |  |
| AMX | Resistant | 4 | 161 | 75% | 0.036 | 0.001 |
|  | Sensitive | 0 | 479 |  |  |  |
| CLR | Resistant | 1527 | 239 | 91.19% | 0.824 | <0.001 |
|  | Sensitive | 48 | 1443 |  |  |  |
| MTZ | Resistant | 581 | 44 | 92.54% | 0.065 | 0.005 |
|  | Sensitive | 3 | 2 |  |  |  |
| LEV | Resistant | 400 | 22 | 95.05% | 0.895 | <0.001 |
|  | Sensitive | 11 | 234 |  |  |  |
